# Evaluating Ecosystem Services Provided by Non-Native Species: An Experimental Test in California Grasslands

**DOI:** 10.1371/journal.pone.0075396

**Published:** 2014-09-15

**Authors:** Claudia Stein, Lauren M. Hallett, W. Stanley Harpole, Katharine N. Suding

**Affiliations:** 1 Environmental Science, Policy & Management, University of California, Berkeley, California, United States of America; 2 Ecology, Evolution and Organismal Biology, Iowa State University, Ames, Iowa, United States of America; Dauphin Island Sea Lab, United States of America

## Abstract

The concept of ecosystem services – the benefits that nature provides to human's society – has gained increasing attention over the past decade. Increasing global abiotic and biotic change, including species invasions, is threatening the secure delivery of these ecosystem services. Efficient evaluation methods of ecosystem services are urgently needed to improve our ability to determine management strategies and restoration goals in face of these new emerging ecosystems. Considering a range of multiple ecosystem functions may be a useful way to determine such strategies. We tested this framework experimentally in California grasslands, where large shifts in species composition have occurred since the late 1700's. We compared a suite of ecosystem functions within one historic native and two non-native species assemblages under different grazing intensities to address how different species assemblages vary in provisioning, regulatory and supporting ecosystem services. Forage production was reduced in one non-native assemblage (medusahead). Cultural ecosystem services, such as native species diversity, were inherently lower in both non-native assemblages, whereas most other services were maintained across grazing intensities. All systems provided similar ecosystem services under the highest grazing intensity treatment, which simulated unsustainable grazing intensity. We suggest that applying a more comprehensive ecosystem framework that considers multiple ecosystem services to evaluate new emerging ecosystems is a valuable tool to determine management goals and how to intervene in a changing ecosystem.

## Introduction

Increasing human influence over Earth's ecosystems has resulted in landscapes with many species assemblages that no longer resemble historic baselines [1], [2]. These changes confront conservationists, land managers and policy makers with questions about how to prioritize management actions, particularly in cases where historic assemblages have changed radically due to widespread species invasion [3], [4].

The ecosystem service framework has arisen as one promising avenue to evaluate effects of changed species assemblages [5], [6], [7]. Species interact with their environment to affect a range of ecosystem functions, such as nutrient cycling and biomass production. When these ecosystem functions affect human wellbeing, they are considered ecosystem services [8]. The ecosystem service framework highlights the breadth of ways in which species can affect human wellbeing, from enhancing culture and providing food to regulating climate conditions. Moreover, it highlights that different ecosystems may provide some, if not all, of the same functions. Thus, the ecosystem service framework can holistically characterize how species affect the environment and provides a way to prioritize landscape management.

The melding of ecosystem services and changed species composition may be nowhere stronger than in California grasslands. While historic records are sparse, California grasslands are thought to have consisted of a range of numerous cool-season perennial grasses, including *Stipa pulchra* Hitchc. (purple needle grass) and *Elymus glaucus* Buckley (wild rye), as well as a diverse set of annual forbs [9]. After European settlement, non-native annual Mediterranean grasses, such as *Avena fatua* L. (wild oats) and *Bromus hordeaceus* L. (soft brome), replaced much of the native grassland. Now covering half of the state, these non-native grasslands provide one of the most important ecosystem services in California, forage for cattle, sustaining a 1.3 billion dollar industry [10], [11].

More recently, a second wave of Mediterranean species, such as *Taeniatherum caput-medusae* L. (Medusahead) and *Centaurea solistitialis* L. (yellow starthistle), is jeopardizing grassland forage potential due to the low palatability of these species [12], [13]. Because of the risks these species pose to the cattle industry, much attention is directed at forage impacts, but our understanding about how changing species assemblages will affect other ecosystem functions or native species conservation is limited.

Thus, California's rangelands are an intriguing model system for challenging questions in invaded landscapes across the globe: How much do different species assemblages vary in their ability to provide forage, as well as other less-monitorized regulatory and supporting services? How do we balance the cultural needs of protecting native species with other ecosystem services? We answer these questions using an experimental approach in which we established replicate plots of native perennial grasses, and two non-native annual grass species assemblages in a common environment. In contrast to observational studies, this approach allowed us to isolate species-specific effects on ecosystem functions by holding abiotic factors, such as soil type and precipitation amount, constant across species assemblages. We implemented a grazing gradient across these assemblages to evaluate which functions will be maintained under increasing grazing intensity. After allowing these treatments time to establish, we measured multiple functions related to a range of ecosystem services outlined by the Millennium Ecosystem Assessment [8]. These included forage potential, the primary provisioning service provided by California grasslands, and native cover and diversity, which are important cultural services for many Californians [14]. Additionally, we measured carbon mineralization, an increasingly-valued regulatory service [15], as well as nitrogen cycling and litter decomposition rates, which are key supporting services in the system.

## Materials and Methods

### Site description

The experiment was located in valley grassland at the University of California Sierra Foothill Research Extension Center (SFREC), Browns Valley, California, USA (39° 15' N, 121° 17' W). The site is characterized by a Mediterranean climate with cool, wet winters and hot, dry summers. Annual rainfall is 700 m, most (>92%) of which falls during the growing season from October through April [16]. The soil in our study area is a loamy, xeric alfisol [17]. The study areas had been grazed by cattle for 150 years prior to our experiment, but we fenced them to facilitate our grazing manipulations over the course of the study.

Like other valley grasslands throughout California, our study sites comprise three primary vegetation types which are characterized by their dominant species: native perennial bunchgrasses (*Stipa pulchra* Hitchc., *Elymus glaucus* Buckley, *Melica californica* Scribner), non-native annual forage grasses (*Avena spp.*, *Bromus hordeaceus* L., *Festuca perennis* L.; hereafter ‘non-native forage grasses’), and non-native invasive weed species such as the annual grass medusahead (*Taeniatherum caput-medusae* L.; hereafter ‘non-native weed’). It is common for these species assemblages to occur separately, both as patches and at the landscape scale. Native dominance is actively maintained through management in some areas, whereas forage grasses tend to dominate elsewhere and invasion by medusahead often result in the formation of monotypic stands. As areas infested by medusahead are avoided by livestock as soon as flowerheads appear [18], grazing capacity can be reduced by up to 75 to 90% [19], [20].

No permits were required for the described study, which complied with all relevant regulations. Endangered or protected species were not involved in this study.

### Creation of different species assemblages along a grazing gradient

In accordance with the naturally dominating vegetation types in our study area, we created three different species assemblages of the aforementioned vegetation types: native perennial bunchgrasses, non-native forage grasses, and the non-native weed (medusahead). We established these assemblages with a mixture of planting perennial plugs, seeding annual species, and weeding in 2007, in 40 m×10 m plots within four replicate blocks, split across two pastures, for a total of 12 species assemblage plots (see Figure S1 for details on the experimental design and set up).

Following the establishment of the species assemblages for two seasons, we split the species assemblage plots in six split-plot treatments representing different levels of grazing intensity in May 2008. The grazing intensity manipulation was achieved with a combination of trampling by cattle and mechanical mowing to establish a grazing gradient with six different grazing levels, ranging from non-grazed to highly grazed, the latter representing unsustainable over-grazing [21] (see Figure S1 for details on the grazing treatments). Thus, the different grazing intensities presented here refer to both trampling and mowing effects. We replicated each grazing treatment twice in each of the four main blocks, resulting in eight 9 m×3 m replicate plots of each treatment combination. Because it was unfeasible to manipulate cattle within a 9 m×3 m plot, we implemented the grazing treatment in an ordered, mirror design (none to high, high to none) that extended across all three species assemblage types (Figure S1). This design is similar to line-source experiments commonly used in agricultural studies [22], best satisfying the joint constraints of large-scale grazing manipulations and species plantings. Here, as our emphasis is on effects of species assemblages, we used only three of the six levels of grazing intensity (none, medium, high) in our analyses, for a total of 72 9 m×8 m plots.

### Species and grazing treatment verification

To assess changes in species abundances we identified all vascular plant species within a permanently marked 1 m^2^ subplot and visually estimated the percent cover of each species at peak standing biomass. Species abundance data were taken in mid May 2008, 2009 and 2010.

We characterized our grazing gradient by residual dry matter (RDM), which is the plant material that remains just before the start of a new growing season and is a standard measure of grazing intensity in rangeland management [21]. We harvested RDM in early October each year by clipping a 0.25×0.25 m subplot within each grazing x species assemblage plot, drying the biomass (60°C for 72 h) and weighing it. To avoid re-harvesting the same area we shifted the subplot position each year.

### Ecosystem service measurements

In 2010, we measured ecosystem functions corresponding to key ecosystem services (forage production, native diversity, soil carbon sequestration and nutrient retention) within each replicate (n = 72) to assess differences among the three species assemblages and in response to grazing (Table 1). We waited until 2010 to allow the established species assemblages to modify the abiotic environment; 2010 also represented an average precipitation year with a total annual precipitation of 641 mm [16].

**Table 1:** Ecosystem service measurements taken in three different grassland ecosystem types.

Ecosystem service	Variable	Measurement
Provisioning service	Forage potential (F)	Aboveground biomass × (relative abundance of palatable species† +0.3‡ × relative abundance of non-palatable species) [g m^−2^]
Cultural services	Native cover (NC)	Abundance of native species [m^−2^]
	Diversity (H)	Shannon's diversity index*
	Invasibility (INV)	Number of naturally establishing species
Regulatory services	Belowground net primary productivity (BNPP)	Fine root production [mg 250 cm^−3^]
	Carbon mineralization (CM)	Potential soil respiration [µmol CO_2_ min^−1^ g^−1^ soil]
Supporting services	Nitrogen cycling (N)	Plant available nitrogen [gN g soil^−1^ day^−1^]
	Decomposition rate (DC)	Potential litter decomposition rate [mg day^−1^]

All measurements were taken in 2010.

† excluded non-palatable species: *Aegilops triunciales* L., *Brachypodium distachyon* (L.) Beauv., *Bromus diandrus* Roth, *Carduus pycnocephalus* L., *Centaurea solistitialis* L., *Taeniatherum caput-medusae* L.

‡ weighting factor to take into account that non-palatable species provide some low quality forage during their early development stages which we estimated to be approx. 30% of their total biomass.

* as number of initially planted species varied among the grassland types, we did not use species richness as a diversity index.

To measure forage production we harvested total aboveground biomass (peak standing biomass plus the biomass removed by each respective mowing treatment) from a randomly-positioned 0.25 m×0.25 m subplot, dried (60°C for 72 h) and weighed it. We corrected this value by the proportion of non-palatable species (see Table 1) in the respective plot. To quantify cultural services we calculated the percent cover of native species and species diversity (Shannon Evenness), including both species that were original planted and those that had naturally established in each plot. We defined invasibility as the number of naturally establishing plant species.

We used two measures of soil carbon sequestration: fine root production and potential soil respiration rates. We measured fine root production using the root ingrowth technique. In December 2009, we filled fiberglass mesh bags (mesh size 1.5 mm, 4 cm diameter, 20 cm height) with root-free sieved soil and vertically buried them 20 cm deep in the rooting zone for 15 weeks. We homogenized the soil in each core and picked roots from the soil cores by hand over a standardized time period. Subsequently, we cleaned the roots of residual soil and detritus, dried them at 60°C to constant mass and weighed them. In February 2010, we measured potential soil respiration rates from freshly collected soil samples (we pooled three subsamples per grazing × species assemblage plot, each 2 cm diameter, 10 cm depth). We placed 2 g of air dried root-free soil into glass vials and added DI water to reach 25% soil moisture. We incubated two replicates from the pooled subsamples for each of the treatment plots and allowed each to reach ambient temperature of approximately 20°C; we opened the vials just prior to the incubation so that headspaces would equilibrate with ambient air. Then we sealed the vials, maintained them in the laboratory at 20°C, and collected 10 mL of headspace after two hours. We immediately injected each gas sample into an EGM-4 Infrared Gas Analyzer.

To characterize nutrient retention, we measured potential decomposition rates and nitrogen availability. We measured potential decomposition rates via litterbags (nylon screen, 2 mm mesh size, 10 cm×17 cm) filled with three pieces of filterpaper (Whatman No. 1, 11 mm diameter) that we placed directly on the soil surface on Dec.13^th^, 2009 and removed on March 30th, 2010. We cleaned the filterpaper of residual soil and detritus, dried it at 60°C to constant mass and weighed it. We quantified soil nitrogen availability using three ion exchange resin bags (Sigma Aldrich Dowex Marathon MR-3 hydrogen and hydroxide form 13687-U mixed beads wrapped in Nylon/Lycra swimsuit liner material) placed in the center of each plot at a depth of 10 cm. We placed the resin bags in the ground from December 16^th^, 2009 to March, 23^rd^, 2010 and extracted them with 2 mol/L KCL. We measured inorganic-N availability (ammonium and nitrate) with a SmartChem autoanalyzer.

### Data analyses

We verified the different species assemblages using constrained correspondence analysis (CCA) on square-root transformed species abundance data from 2008–2010 (cca function in the vegan library in R v2.15.1). Pasture and year were included as covariates. Rare species, i.e. species only occurring in one or two samples during the three years, were excluded from the analysis [23].

To quantify how ecosystem functions differed among the three ecosystem types under different grazing intensities we used a linear mixed effect model (proc MIXED, SAS version 9.2, SAS Institute, Cary, N.C.) with ecosystem type, grazing intensity, direction of the grazing gradient (replicate of grazing within a species assemblage, see Figure S1 for details) and their interaction as fixed factors. Pasture, block within pasture, and the interaction of block within pasture and position as well as with grazing intensity level were used as random effects. A ‘Toeplitz’ (3-banded) variance/covariance matrix was used to account for the spatial dependency of the sample units [24], and residuals were approximately normal (see Figure S2). We used log transformation for RDM, forage potential, fine root production, resin available nitrogen, and a square root transformation for potential litter decomposition and soil respiration rates.

## Results

**Figure 1:**
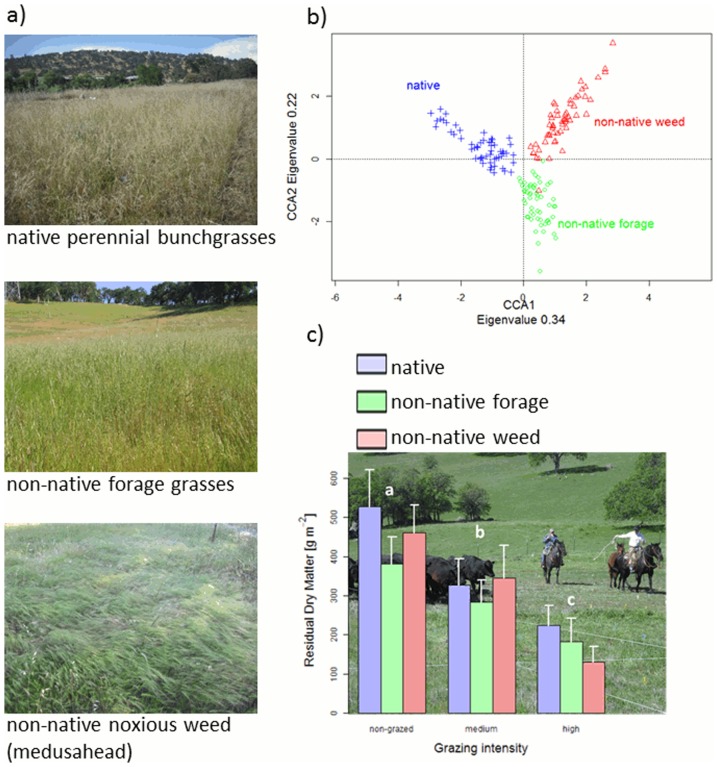
The three grassland species assemblages investigated in this study. The (a) three original planted species assemblages are clearly distinguishable based on their species composition as shown by (b) results of CCA of species abundances from 2008–2010 with year and study site as covariate. (c) Residual dry matter measured in 2010 to describe grazing gradient within the three ecosystem types. Letter above bars indicate significant differences among grazing levels as determined by planned orthogonal contrasts at P<0.01.

We successfully established three distinct species assemblages (Figure 1a), which all showed a similar reduction in RDM due to the grazing treatments (Figure 1b, Table S1). Medium and high grazing intensity reduced RDM by 34% and by 67% compared to non-grazed conditions, which yielded an average RDM of 401+110 g m^−2^ (back transformed least square mean +95% CI).

### Provisioning service

**Figure 2:**
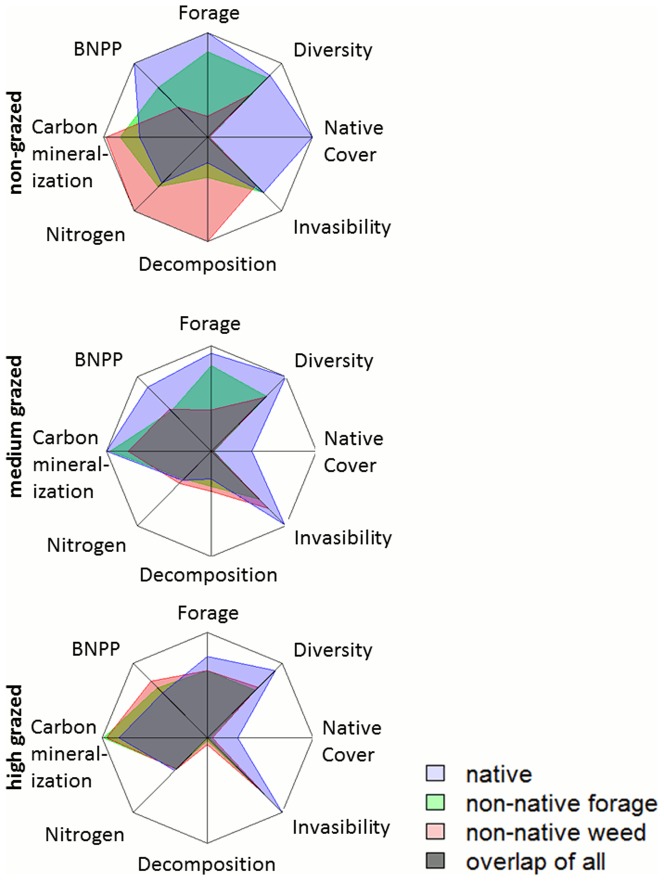
Ecosystem services provisioned by each species assemblage along a grazing gradient. Ecosystem services are scaled by maximum and minimum observed values (using back transformed least square means). The outside of the web corresponds to maximum service provisioning. Explanations of ecosystem services are given in Table 1.

Forage potential of the three species assemblages was differently affected by the grazing treatments (Table S1 & S2, significant grazing × ecosystem type interaction P<0.001): within both non-native species assemblages, forage potential responded positively to grazing (Figure 2). In the non-native weed assemblage, available forage was two to three times higher under medium and high grazing intensity compared to non-grazed conditions, respectively. In contrast, forage potential within the native assemblage did not respond to the grazing treatment, but the native assemblage delivered in general the most consistent forage source, with forage potential equal or greater to the non-native assemblages (Figure 2).

### Cultural services

Native species were rare within both non-native species assemblages, with a mean total cover of less than 2%. Within the native assemblage, native cover decreased due to grazing (Table S1, significant vegetation × grazing interaction P<0.001). In the non-grazed plots, native species reached a mean abundance of 57+5% (back transformed least square mean +95%CI), and decreased by 62% and 71% under medium and high grazing, respectively. Native species were mainly replaced by naturally establishing exotic forb species, such as legume species (*Trifolium* spp.), and rosette forming Asteraceae (*Hypericum* spp., *Leontodon* spp.) or *Erodium* spp.

Diversity did not significantly differ among the three species assemblages and did not change in response to grazing (Table S1 & S2).

### Regulatory services

The effect of grazing on fine root production was dependent on the resident species assemblages (Table S1, significant vegetation × grazing interaction, P<0.001). Within the native ecosystem, fine root production decreased by 11% and 44% due to medium and high grazing, respectively (Table S2). In contrast, a positive grazing effect was detectable within the non-native weed assemblage, with mean fine root production increasing by 40% and 160% at medium and high grazing, respectively.

Potential soil respiration rates did not differ among the three species assemblages and did not change in response to grazing (Table S1 & S2).

Non-added species naturally established in the experiment and overall invisibility did not differ among the three species assemblages and did not change in response to grazing (Table S1 & S2).

### Supporting services

Nitrogen availability decreased by 55% under grazing compared to non-grazed conditions within all species assemblages, but no differences were detected among the three species assemblages (Table S1 & S2). Potential litter decomposition rates were significantly reduced due to the grazing treatment within all species assemblages (Table S1), and decreased by 35% and 94% due to medium and high grazing, respectively (Table S2). There was a strong trend that decomposition rates were almost twice as fast within the non-native weed assemblage compared to the native and the non-native forage assemblages, however, this was only marginal significant (P = 0.08).

## Discussion

Using California grassland ecosystems as a model system, our study demonstrates changed species assemblages may alter some ecosystem functions while maintaining others. Ecosystem services tied to key species, such as cultural appreciation of native species, were inherently lower in both non-native dominated systems, whereas regulatory and supporting services, such as carbon storage and nutrient cycling, were similar regardless of the dominant type of grass species (Figure 2). Provisioning services such as forage potential, which are of major interest in these grassland systems, were maintained in one non-native system but not in another. High grazing intensity caused all ecosystems to become similar in the provisioning of ecosystem services (Figure 2).

As species assemblages shift and adapt to environmental change or land use, there may be instances in which managing for the provisioning of certain ecosystem services provides a valuable management framework [25]. For example, the functional convergence between the native and non-native assemblages under high grazing was related to a grazing-induced species convergence: across ecosystems, grazing led to an increase in annual forbs (e.g. *Erodium* spp., *Trifolium* spp.). However, the native perennial grasses were more sensitive to grazing than annual grasses and may be less likely to persist under high grazing intensities. Along another axis, forage production actually increased with grazing in the non-native weed (medusahead) dominated grasslands due to the replacement of medusahead with more palatable annual forb species. Thus, our result highlights the importance of tailoring management actions to specific ecosystem types, as the same strategy might cause opposite effects within different ecosystems.

It is a tautology that cultural services tied to native species are necessarily low in non-native ecosystems. Therefore it is of immense importance to clearly set management priorities. If native species conservation is the primary goal, management decisions need to be shaped around that goal. However, alone the ongoing debate about the effects of grazing management on native species conservation shows that results are often controversial [26], [27].

Evaluating ecosystems based on specific services requires determining priorities and understanding that trade-offs may be inevitable. For example, clear differences existed among our three species assemblages with respect to native species conservation and forage production. While these services are often the main goals in rangeland management, regulatory services such as carbon sequestration are gaining prominence in how managers evaluate systems, and supporting services such as nutrient retention play key roles in maintaining many valued services. How desirable ecosystem services will be evaluated is context dependent making generalizations difficult [28]. For instance, lower carbon mineralization and nutrient cycling rates might be desirable in our study system as they reduce potential carbon and nitrogen loss rates.

We suggest that applying a more comprehensive ecosystem framework to evaluate new emerging ecosystems is a valuable tool to inform management goals across species assemblages and to decide if or how to intervene in a changing ecosystem [29]. As our understanding of the similarities and differences between ecosystems that differ in their species composition extends to cover many ecosystem functions, holistic displays (e.g., Figure 2) that compare various ecosystem services and ecosystem types can help management priorities and critical decision making points. They are also useful tools to monitor the progress of restoration or management efforts [30] and indicate which parts of the system are the most sensitive to certain management actions, as we demonstrate here for grazing intensity.

## Supporting Information

Figure S1 **Experimental design within one of the two replicate pastures.** We established three species assemblages, which are common across rangelands in California, consisting of native perennial bunchgrasses (*Stipa pulchra* Hitchc., *Elymus glaucus* Buckley, *Melica californica* Scribner representing one approximation of a historical (native) ecosystem, non-native annual forage grasses (*Avena fatua* L., *Avena barbata* Link, *Bromus hordeaceus* L., *Festuca perennis* L.) representing a non-native species assemblage of high forage value, and a non-native weed, medusahead (*Taeniatherum caput-medusae* L.) representing a very recently emerged noxious weed-dominated ecosystem. In October 2006, we planted two 40 m×10 m areas of each of the three species assemblages in two pastures. Replicates of species assemblages were set up in two blocks per pasture and their location was randomized within blocks. Both pastures were fenced to exclude large herbivores. Soil solarization, a common method in agriculture for controlling soil-borne pathogens and weeds [31], was applied as a pre-planting soil treatment. We tilled the soil, and then covered the soil with black Polyethylene plastic tarps for 14 days, trapping solar heat to reduce seed bank [32]. The native grasses were planted at a density of 12.5 plugs m^−2^ with equal numbers for the three species (total 20,000 plugs, plug diameter 2.5–6 cm, Hedgerow Farms, Winters, California, USA). The annual forage grasses were seeded at equal rates with a total rate of 23–28 g seeds per species m^−2^ (Pacific Coast Seed, Livermore, California, USA) and *T. caput-medusae* was seeded at a rate of 21 g seeds m^−2^ (seeds collected at the research center). Plots were watered and broadleaf herbicides were applied only during the first growing season to ensure successful and even establishment of the species assemblages. We did not manipulate species composition or resource availability in subsequent years. We initiated the grazing treatments after 1.5 years to allow establishment of the vegetation. We split each of the four vegetation blocks in half, and in each half, applied a gradient of grazing treatments laid out perpendicular to the species assemblage treatments in 3×30 m strips with an additional 2 m buffer zone on each side of the species assemblages blocks. For this study we used the following three of the six grazing intensity levels: 1) control that was not trampled or mowed (“non-grazed”); 2) medium grazed, which was trampled twice per year (in late March when plants started flowering and in June/July after most plants were senescent) with one mowing treatment right before the late March trampling treatment; and 3) high grazed, which was trampled twice a year as treatment two with mowing prior to each trampling and an additional mowing in late February during early plant growth. The mowing treatment included cutting the vegetation at a height of 2 cm above ground with a mechanical “walk-behind” mower and removing the biomass. For each trampling treatment 40–42 cattle (up to one year old, Black Angus Mix, 900–1000lbs/cattle) were herded onto the respective strips for 30–45 minutes depending on soil conditions. Under moist soil conditions, trampling times were shortened to achieve comparable trampling effects compared to dry soil conditions. In order to have areas large enough for cattle, trampling treatments encompassed all species assemblages simultaneously as well as same grazing intensity levels (one fenced area); cattle were not allowed to graze to avoid uncontrolled biomass removal. Thus, the experimental design was a split plot arrangement in which the subplot levels (grazing intensity) represent a non-randomized division of the species assemblages (whole plots). Each plot representing a specific species assemblage × grazing intensity combination was 3 m×10 m in size. We acknowledge several levels of dependence within our design: within pasture, within grazing gradient, and spatially proximity of adjacent levels of grazing treatment. However, we feel that this design, which derived from line-source experiments often used in agricultural studies [22], best enabled the joint constraints of larger scale grazing treatments with logistical planting restrictions in our experimental manipulations. Using a ‘Toeplitz’ (3-banded) variance/covariance matrix enables us to account for the spatial dependency of the sample units [24], i.e. we can fit variance models to structures at the residual (R) and the random (G) level.
(TIF)

Figure S2 **Plot of the residuals for the linear mixed effect model analyses of Shannon diversity.**
(DOCX)

Table S1 **Results for main effects of linear mixed effect models (F-values, t-values for orthogonal contrasts‡) of the effects of manipulating grazing intensity on ecosystem functions measured in three different experimentally established species assemblages in California grasslands.**
(DOCX)

Table S2 **Mean and range of ecosystem services measured in three experimentally established species assemblages in California grasslands in response to manipulation of grazing intensity.**
(DOCX)
