# Supplementary figures and images for: Evaluating Ecosystem Services Provided by Non-Native Species: An Experimental Test in California Grasslands
Source: PLoS One. 2014 Sep 15;9(9):e75396. doi: 10.1371/journal.pone.0075396 (PMC4164352; doi:10.1371/journal.pone.0075396)

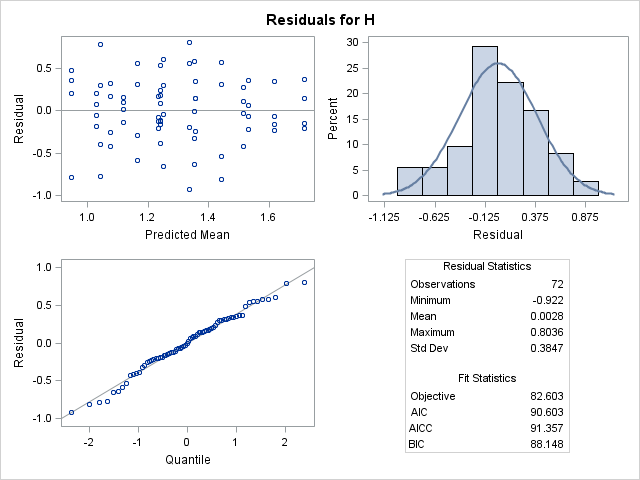


Figure S2: Plot of the residuals for the linear mixed effect model analyses of Shannon diversity.

Supplement: Figure S2 — Plot of the residuals for the linear mixed effect model analyses of Shannon diversity. (DOCX) [file pone.0075396.s002.docx]
