# Supplementary material for: Evaluating Ecosystem Services Provided by Non-Native Species: An Experimental Test in California Grasslands
Source: PLoS One. 2014 Sep 15;9(9):e75396. doi: 10.1371/journal.pone.0075396 (PMC4164352; doi:10.1371/journal.pone.0075396)
Supplement: Table S1 — Results for main effects of linear mixed effect models (F-values, t-values for orthogonal contrasts‡) of the effects of manipulating grazing intensity on ecosystem functions measured in three different experimentally established species assemblages in California grasslands. (DOCX) [file pone.0075396.s003.docx]

**Table S1:** **Results for main effects of linear mixed effect models (F-values, t-values for orthogonal contrasts‡) of the effects of manipulating grazing intensity on ecosystem services measured in three experimentally established species assemblages in California grasslands.**

| Source of variation | | *df* | Residual dry matter | | Forage production | | Diversity | | Native cover | | Invasibility | | Belowground net primary productivity | | De-composition | | C-mineral-ization | | Nitrogen | |
| --- | --- | --- | --- | --- | --- | --- | --- | --- | --- | --- | --- | --- | --- | --- | --- | --- | --- | --- | --- | --- |
| Species assemblage | | 2 (6) | 0.47 |  | 7.06 | (✝) | 2.40 |  | 86.91 | *** | 2.73 |  | 2.99 |  | 3.70 |  | 0.30 |  | 0.54 |  |
|  | native *vs* non-native | 1 |  |  | 2.45 | (✝) |  |  | 13.18 | *** |  |  |  |  |  |  |  |  |  |  |
|  | forage *vs* medusa | 1 |  |  | 2.85 | (✝) |  |  | -0.32 |  |  |  |  |  |  |  |  |  |  |  |
| Direction | | 1 (3) | 0.37 |  | 1.81 |  | 0.30 |  | 0.02 |  | 0.18 |  | 0.11 |  | 0.05 |  | 1.80 |  | 0 |  |
| Grazing | | 2 (6) | 37.60 | ** | 1.04 |  | 0.70 |  | 14.85 | * | 0.81 |  | 0.36 |  | 13.99 | * | 1.60 |  | 8.72 | * |
|  | non-grazed *vs* grazed | 1 | 15.33 | ** |  |  |  |  | 5.45 | ** |  |  |  |  | 3.74 | * |  |  | 4.16 | * |
|  | medium *vs* high | 1 | 11.84 | ** |  |  |  |  | 0.14 |  |  |  |  |  | 3.99 | * |  |  | -0.40 |  |
| Species ass. x position | | 2 (40) | 1.74 |  | 2.10 |  | 0.95 |  | 3.59 |  | 2.12 |  | 3.40 |  | 0.33 |  | 0.23 |  | 1.58 |  |
| Grazing x direction | | 2 (40) | 2.51 |  | 0.72 |  | 0.10 |  | 0.75 |  | 1.54 |  | 0.97 |  | 3.92 | (✝) | 2.82 |  | 0.03 |  |
| Species ass. x grazing | | 4 (40) | 1.74 |  | 10.31 | *** | 0.80 |  | 29.42 | *** | 1.44 |  | 6.31 | ** | 0.99 |  | 2.10 |  | 0.60 |  |
|  | native *vs* non-native x non-grazed *vs* grazed | 1 |  |  | -3.07 | * |  |  | -10.5 | ** |  |  | -2.32 | (✝) |  |  |  |  |  |  |
|  | native *vs* non-native x medium *vs* high | 1 |  |  | -1.60 |  |  |  | -2.48 | (✝) |  |  | -3.13 | * |  |  |  |  |  |  |
|  | forage *vs* weed x non-grazed *vs* grazed | 1 |  |  | 5.00 | *** |  |  | 0.86 |  |  |  | 3.46 | * |  |  |  |  |  |  |
|  | forage *vs* weed x medium *vs* high | 1 |  |  | 3.35 | * |  |  | 1.51 |  |  |  | 0.02 |  |  |  |  |  |  |  |

Results are shown for main effects from linear mixed effect models (F-values, t-values for orthogonal contrasts‡). Significance levels are given as * P<0.05, **P<0.01, ***P<0.001, (✝) non significant after accounting for multiple comparisons with the Benjamini–Hochberg method (line-by-line) [1]. Ecosystem services are explained in Table 1. Direction refers to the location of the grazing gradient within an experimentally planted species assemblage (see Figure S1 for details).

‡ Note:

We used the following orthogonal contrasts to test for significant differences among species assemblages: (1) “native *vs.* non-native” compared the historical native vegetation to both non-native assemblages; (2) “forage vs. weed” tested for differences between the two non-native species assemblages; (3) “non-grazed *vs.* grazed” compared the control to both the medium and the high grazing treatment; and (4) “medium *vs.* high” tested for differences in grazing intensity. If significant species assemblage × grazing interactions occurred we performed the following orthogonal contrasts within the interaction only: (5) “native *vs.* non-native × non-grazed *vs.* grazed” and (6) “native *vs.* non-native × medium *vs.* high” to test if the historical native vegetation responded differently to the grazing treatments compared to both non-native species assemblages; and (7) “forage vs. weed × non-grazed *vs.* grazed” and (8) “forage vs. weed × medium *vs.* high” to test if the two non-native species assemblages differed in their response to the grazing treatments.

REFERENCES

1. Verhoeven KJF, Simonsen KL, McIntyre LM (2005) Implementing false discovery rate control: increasing your power. Oikos 108:643-647.
