# Supplementary material for: Evaluating Ecosystem Services Provided by Non-Native Species: An Experimental Test in California Grasslands
Source: PLoS One. 2014 Sep 15;9(9):e75396. doi: 10.1371/journal.pone.0075396 (PMC4164352; doi:10.1371/journal.pone.0075396)
Supplement: Table S2 — Mean and range of ecosystem services measured in three experimentally established species assemblages in California grasslands in response to manipulation of grazing intensity. (DOCX) [file pone.0075396.s004.docx]

**Table S2:** **Mean and range of ecosystem services measured in three experimentally established species assemblages in California grasslands in response to manipulation of grazing intensity.**

|  |  | Forage potential  [g m^-2^] | | Native Cover  [%] | | Shannon Diversity | | Invasibility [species m^-2^] | | Below- ground net primary productivity  [mg 250cm^-3^] | | De-composition  [mg day^-1^] | | C-mineralization  [µmol CO_2_ min^-1^ g^-1^ soil] | | Nitrogen [gN g soil^-1^ day^-1^] | |
| --- | --- | --- | --- | --- | --- | --- | --- | --- | --- | --- | --- | --- | --- | --- | --- | --- | --- |
| Species assemblages | grazing intensity | Mean | | Mean | | Mean | | Mean | | Mean | | Mean | | Mean | | Mean | |
|  |  | Min-max | | Min-max | | Min-max | | Min-max | | Min-max | | Min-max | | Min-max | | Min-max | |
| native | non-grazed | 383.4 | | 58.7 | | 1.4 | | 11.4 | | 58.1 | | 14.5 | | 201.5 | | 0.7 | |
|  |  | 143.7 | 802.3 | 41.2 | 74.0 | 0.6 | 2.0 | 5.0 | 19.0 | 43.6 | 77.3 | 0.6 | 51.0 | 61.5 | 378.2 | 0.0 | 2.1 |
| native | medium | 328.4 | | 22.3 | | 1.7 | | 14.1 | | 49.1 | | 16.4 | | 311.3 | | 0.2 | |
|  |  | 220.5 | 468.1 | 14.5 | 33.5 | 1.4 | 2.1 | 11.0 | 19.0 | 35.4 | 65.3 | 0.0 | 72.8 | 72.1 | 501.9 | 0.0 | 0.4 |
| native | high | 300.2 | | 16.5 | | 1.5 | | 12.9 | | 34.7 | | 0.6 | | 272.2 | | 0.4 | |
|  |  | 172.6 | 458.5 | 10.5 | 24.5 | 1.1 | 1.9 | 9.0 | 16.0 | 11.0 | 59.1 | 0.0 | 2.8 | 91.7 | 467.3 | 0.1 | 1.0 |
| non-native forage | non-grazed | 267.6 | | 1.1 | | 1.3 | | 10.3 | | 39.2 | | 19.3 | | 246.7 | | 0.8 | |
|  |  | 69.7 | 495.7 | 0.0 | 1.7 | 0.4 | 2.1 | 7.0 | 14.0 | 26.8 | 54.1 | 0.4 | 59.2 | 124.8 | 463.5 | 0.1 | 4.1 |
| non-native forage | medium | 371.7 | | 1.9 | | 1.2 | | 9.1 | | 33.0 | | 21.2 | | 297.0 | | 0.2 | |
|  |  | 122.6 | 959.6 | 0.0 | 4.5 | 0.9 | 1.8 | 4.0 | 14.0 | 16.4 | 76.3 | 0.7 | 92.1 | 68.0 | 632.3 | 0.1 | 0.3 |
| non-native forage | high | 375.4 | | 1.6 | | 1.1 | | 8.6 | | 39.1 | | 2.5 | | 314.9 | | 0.3 | |
|  |  | 89.8 | 882.1 | 0.0 | 4.0 | 0.8 | 1.3 | 4.0 | 14.0 | 24.4 | 77.2 | 0.0 | 15.1 | 158.0 | 605.0 | 0.0 | 0.7 |
| non-native weed | non-grazed | 27.1 | | 1.6 | | 1.1 | | 8.7 | | 21.5 | | 39.1 | | 300.3 | | 1.3 | |
|  |  | 13.1 | 52.8 | 0.0 | 4.0 | 0.3 | 1.8 | 4.0 | 14.0 | 4.9 | 52.5 | 10.7 | 96.1 | 167.3 | 540.2 | 0.3 | 2.4 |
| non-native weed | medium | 116.1 | | 1.6 | | 1.2 | | 10.5 | | 32.0 | | 24.8 | | 245.0 | | 0.3 | |
|  |  | 20.1 | 249.7 | 0.0 | 7.6 | 0.6 | 1.9 | 6.0 | 15.0 | 16.2 | 66.5 | 0.0 | 81.6 | 156.4 | 412.1 | 0.2 | 0.8 |
| non-native weed | high | 312.8 | | 3.1 | | 1.1 | | 10.8 | | 43.4 | | 8.6 | | 283.0 | | 0.3 | |
|  |  | 62.1 | 632.0 | 0.5 | 8.5 | 0.7 | 1.5 | 6.0 | 15.0 | 26.7 | 70.7 | 0.0 | 43.9 | 105.8 | 534.0 | 0.1 | 0.5 |
